# Supplementary material for: Gjd2b-mediated gap junctions promote glutamatergic synapse formation and dendritic elaboration in Purkinje neurons
Source: eLife. 2021 Aug 4;10:e68124. doi: 10.7554/eLife.68124 (PMC8382294; doi:10.7554/eLife.68124)
Supplement: Supplementary file 1. [file elife-68124-supp1.docx]

Mean synapse density of wildtype and gjd2b^-/-^ larvae

| **Genotype** | **Animal** | **Mean synapse density (per cu. micron)** |
| --- | --- | --- |
| gjd2b^-/-^ | A1 | 9.6 ± 5.2 |
| gjd2b^-/-^ | A2 | 13.1 ± 4.7 |
| gjd2b^-/-^ | A3 | 10.6 ± 4.5 |
| WT | B1 | 13.5 ± 5.4 |
| WT | B2 | 14.1 ± 4.8 |
| WT | B3 | 14 ± 5.1 |
