# Supplementary material for: Gjd2b-mediated gap junctions promote glutamatergic synapse formation and dendritic elaboration in Purkinje neurons
Source: eLife. 2021 Aug 4;10:e68124. doi: 10.7554/eLife.68124 (PMC8382294; doi:10.7554/eLife.68124)
Supplement: Supplementary file 3. [file elife-68124-supp3.docx]

**Statistical Summary Table**

**Figure 1:**

| **Figure number** | **Statistical test** | **Comparison** | **F/U value** | **N** | **p-value** |
| --- | --- | --- | --- | --- | --- |
| Figure 1C | Mann-Whitney | Inter event interval (WT-SBMO) | 37285 | Control=7 cells from 7 larvae  SBMO=12 cells from 12 larvae | <0.0001 |
| Figure 1D | Mann-Whitney | Peak Amplitude (WT-SBMO) | 25537 | Control=7 cells from 7 larvae  SBMO=12 cells from 12 larvae | 0.001532 |
| Figure 1E | Mann-Whitney | Rise time  (WT-SBMO) | 26831 | Control=7 cells from 7 larvae  SBMO=12 cells from 12 larvae | 0.1834 |
| Figure 1F | Mann-Whitney | Decay tau (WT-SBMO) | 25018 | Control=7 cells from 7 larvae  SBMO=12 cells from 12 larvae | 0.01392 |

**Figure 2:**

| Figure 2F | Mann-Whitney | Normalised gjd2b staining intensity (WT-Mutant) | 143 | WT=26 images  Mutant=30 images | <0.001 |
| --- | --- | --- | --- | --- | --- |

**Figure 3:**

| Figure 3C | Mann-Whitney | Inter event interval (WT-Mutant) | 4906 | WT=8 cells  Mutant=10 cells | 0.01977 |
| --- | --- | --- | --- | --- | --- |
| Figure 3D | Mann-Whitney | Peak Amplitude (WT-Mutant) | 3385 | WT=8 cells  Mutant=10 cells | <0.0001 |
| Figure 3E | Mann-Whitney | Rise time  (WT-Mutant) | 2322 | WT=8 cells  Mutant=10 cells | <0.0001 |
| Figure 3F | Mann-Whitney | Decay tau (WT-Mutant) | 2791 | WT=8 cells  Mutant=10 cells | <0.0001 |

**Figure 4:**

| Figure 4E | ANOVA | PPR (WT-SBMO) | 3.979 | Control=5 cells from 5 larvae  SMBO=5 cells from 5 larvae | 0.081 |
| --- | --- | --- | --- | --- | --- |
| Figure 4G | ANOVA | PPR (WT-Mutant) | 2.039 | WT=14 cells from 5 fish  Mutant=13 cells from 4 fish | 0.17 |

**Figure 5:**

| Figure 5B, E | Mann-Whitney | Synaptic density | 86268 | WT= 106 micrographs from 3 larvae  Mutant= 106 micrographs from 3 larvae | <0.0001 |
| --- | --- | --- | --- | --- | --- |
| Figure 5D, F | Mann-Whitney | Synapse maturity | 4922 | WT= 106 micrographs from 3 larvae  Mutant= 106 micrographs from 3 larvae | 0.1194 |

**Figure 6:**

| Figure 6C | Generalised linear model | TDBL (WT-Mutant) |  | WT(5)=50 cells Mutant(5)=27 cells  WT(6)=53 cells Mutant(6)=32 cells  WT(7)=44 cells Mutant(7)=30 cells  WT(8)=36 cells Mutant(8)=44 cells | 0.0484 | |
| --- | --- | --- | --- | --- | --- | --- |
|  | Generalised linear model | TDBL (5dpf-6dpf) |  |  | | 0.0021 |
|  | Generalised linear model | TDBL (6dpf-7dpf) |  |  | | 0.1821 |
|  | Generalised linear model | TDBL (7dpf-8dpf) |  |  | | 0.9845 |
| Figure 6D | Linear model | Hourly growth rate (WT-Mutant) |  | WT=10 cells Mutant=10 cells | | 0.00597 |
| Figure 6E | Linear model | Branch elongation (WT-Mutant) |  |  | | 0.0221 |
| Figure 6F | Linear model | Branch retraction (WT-Mutant) |  |  | | 0.185 |

**Figure 7:**

| Figure 7B | ANOVA | TDBL (Mutant-gjd2b rescue) |  | WT=44 cells Mutant=30 cells Rescue=16 cells Pore dead=18 cells | <0.0001 |
| --- | --- | --- | --- | --- | --- |
|  | ANOVA | TDBL (Mutant-pore dead) |  |  | 0.4370 |
| Figure 7C | Mann-Whitney | Change in branch length elongation (WT-Mutant) | 3779.5 | WT=7 cells Mutant=7 cells | 0.02246 |
|  | Mann-Whitney | Change in branch length retraction (WT-Mutant) | 4048 |  | 0.09797 |

**Supplementary Figures:**

| Figure 1-figure supplement 2 | Mann-Whitney | Normalised gjd2b staining intensity (Uninjected-SBMO) |  | Uninjected=42 images  SBMO=48 images | <0.001 |
| --- | --- | --- | --- | --- | --- |
| Figure 1-figure supplement 2 | Mann-Whitney | Normalised gjd2b staining intensity (Uninjected-Control) |  | Uninjected=30 images  Control=44 images | 0.14 |
| Figure 3-figure supplement 1 | Mann-Whitney | Input resistance in K-gluconate |  | WT = 21 cells  Mutant = 22 cells | 0.62 |
| Figure 3-figure supplement 1 | Mann-Whitney | Input resistance in Cs-gluconate |  | WT = 12 cells  Mutant = 10 cells | 0.02 |
| Figure 6 – figure supplement 1 | Generalised linear model | Soma diameter (WT-Mutant) |  | WT=44 cells  Mutant=30 cells | 0.0540 |
| Figure 6 – figure supplement 1 | Linear mixed model | Number of intersections/micron (WT-Mutant) |  | WT=43 cells  Mutant=29 cells | 0.9086 |
| Figure 6 – figure supplement 1 | Generalised linear model | TDBN (WT-Mutant) |  | WT(5)=50 cells Mutant(5)=27 cells  WT(6)=53 cells Mutant(6)=32 cells  WT(7)=44 cells Mutant(7)=30 cells  WT(8)=36 cells Mutant(8)=44 cells | 0.0002 |
|  | Generalised linear model | TDBN (5dpf-6dpf) |  |  | 0.0797 |
|  | Generalised linear model | TDBN (6dpf-7dpf) |  |  | 0.0123 |
|  | Generalised linear model | TDBN (7dpf-8dpf) |  |  | 0.6475 |
