# Supplementary material for: Gjd2b-mediated gap junctions promote glutamatergic synapse formation and dendritic elaboration in Purkinje neurons
Source: eLife. 2021 Aug 4;10:e68124. doi: 10.7554/eLife.68124 (PMC8382294; doi:10.7554/eLife.68124)
Supplement: Supplementary file 4. [file elife-68124-supp4.docx]

**Summary of experimental manipulations, caveats, controls and conclusions.**

| **Experiments** | **Advantages** | **Caveats** | **Controls for caveats** | **Conclusion** |
| --- | --- | --- | --- | --- |
| **Morphants** | - Quick and efficient gene knockdown approach.  - Provides data lead to further work | - Morpholinos may have non-specific and off-target effects  - Gene knockdowns are transient, incomplete and mosaically knockdown across cells and tissues of organism)  - Hence, the effect on phenotypes is prone to variability | -Specificity tested by the use of a control morpholino with a 5 base mismatch to the target Gjd2b sequence. Control morpholino did not result in Gjd2b knock down (Figure 1 – figure supplement 2).  - Variability and mosaicism addressed by the generation of global knockout of Gjd2b. | The splice block morpholino used in this study effectively and specifically reduces Gjd2b levels. Yet, mutants are needed to address variability and mosaicism. |
| **Mutants** | - mutations in nucleotide sequence verified  - Verified coding sequence frame-shift on mRNA transcript  -Verified that the Gjd2b protein levels are also reduced.  -Ubiquitous, stable and permanent | - Antibody for Gjd2b non-specifically labels another orthologue, Gjd2a  - Hence, only a reduction in Gjd2 expression is noticed rather than a complete lack of signal in IHC of Gjd2b mutants using antibody against perch Cx35 /36. | Use of TALENs for genome-editing ensures specificity due to requirement of two binding sites. | A point mutation in Gjd2b was generated, validated and confirmed by sequencing. |
| **Recording of AMPAR mEPSCs** | - functional readout - Specific to PNs - Specific to glutamatergic synapses | mEPSC frequency may be affected by changes in  - Synapse number  -Probability of vesicle release  - Percent silent synapses. | PPR experiments in morphants and mutants at CF-PN synapses show that probability of release is not altered.  PNs do not seem to possess any NMDAR currents ruling out silent synapses | Highly suggestive of a decrease in the number of glutamatergic synapses impinging on PNs. |
| **Transmission Electron Microscopy** | - Definitive identification of synapses using ultrastructural features resulting in reliable counts.  - Synapses could be classified as mature or nascent based on architecture. | - Counts were done in molecular layer of the cerebellum, but synapses cannot be conclusively assigned to PNs. - Inhibitory and excitatory synapses could not be unequivocally distinguished. | -PNs contribute significantly to the molecular layer synapses as they have an elaborate arbor. Thus a significant proportion of the synapses we counted most likely belong to PNs. | Highly suggestive of a decrease in the number of synapses impinging on PNs. |
| **PN dendritic imaging** | - Daily imaging shows overall growth trends  - Hourly imaging reveals dynamics of elongations and retractions  -Puncta imaging shows relationship of puncta to branch behavior | -Limited to 4 days of imaging due to phototoxicity and embedding stress to larvae. |  | Mutants have stunted dendritic arbors caused by decreased rate of elongations. Gjd2b puncta on branches promote elongation. |
| **Gjd2b rescue in PNs** | - Full length and N-terminal deleted Gjd2b expressed in single isolated PNs. | Whether the expressed Gjd2b forms a docked connexon is not known. | Rescue of dendritic arbor size with full length Gjd2b, but not with the N-terminal deleted version suggests a true functional role for Gjd2b in dendritic growth. | Suggests that Gjd2b could regulate dendrite growth in a cell-autonomous fashion. |
| **Droplet digital PCR of CamKII** | -Quantitative analysis of all camk2 isoforms in whole larval brain from 4 biological replicates | - Measures trancript levels only.  - Global brain levels, not limited to PNs |  | Suggests a decrease in the expression levels of all isoforms of camk2 after Gjd2b is lost. |
| **CaMKII inhibitor treatment** | -Direct pharmacological inhibition of CaMKII to assess effects on PN dendrite elaboration | -Specificity of KN93 to CaMKII  -Drugs were applied to whole animals. | Rescue of dendrite growth was not observed with inactive analog KN92 or with vehicle only. | - Suggests that Gjd2b could regulate dendrite elongation by regulating the expression level and activity of CaMKII. |
